# COVID-19 Infection Process in Italy and Spain: Are Data Talking? Evidence From ARMA and Vector Autoregression Models

**DOI:** 10.3389/fpubh.2020.550602

**Published:** 2020-11-23

**Authors:** Paloma Monllor, Zhenyu Su, Laura Gabrielli, Paloma Taltavull de La Paz

**Affiliations:** ^1^Grupo de Investigación en Economia y Vivienda, Economy and Housing Research group, University of Alicante, Alicante, Spain; ^2^Department of Physiology, Faculty of Medicine, Instituto de Investigación Sanitaria de Valencia - Valencian's Health Research Institute, University of Valencia, Valencia, Spain; ^3^IBSS, Xi'an Jiaotong-Liverpool University, Suzhou, China; ^4^Department of Architecture, IUAV University of Venice, Venezia, Italy; ^5^Department of Urban Studies and Planning, Massachusetts Institute of Technology (MIT), Cambridge, MA, United States

**Keywords:** COVID-19, forecast, ARMA model, vector-autorregression, Italy, Spain, ICU-beds

## Abstract

COVID-19 (coronavirus disease 2019) has spread successfully worldwide in a matter of weeks. After the example of China, all the affected countries are taking hard-confinement measures to control the infection and to gain some time to reduce the significant amount of cases that arrive at the hospital. Although the measures in China reduced the percentages of new cases, this is not seen in other countries that have taken similar measures, such as Italy and Spain. After the first weeks, the worry was whether or not the healthcare system would collapse rather than its response to the patient's needs who are infected and require hospitalization. Using China as a mirror of what could happen in our countries and with the data available, we calculated a model that forecasts the peak of the curve of infection, hospitalization, and ICU bed numbers. We aimed to review the patterns of spread of the virus in the two countries and their regions, looking for similarities that reflect the existence of a typical path in this expansive virulence and the effects of the intervention of the authorities with drastic isolation measures, to contain the outbreak. A model based on Autorregressive and moving average models (ARMA) methodology and including Chinese disease pattern as a proxy, predicts the contagious pattern robustly. Based on the prediction, the hospitalization and intensive care unit (ICU) requirements were also calculated. Results suggest a reduction in the speed of contagion during April in both countries, earlier in Spain than in Italy. The forecast advanced a significant increase in the ICU needs for Spain surpassing 8,000 units by the end of April, but for Italy, ICU needs would decrease in the same period, according to the model. We present the following predictions to inform political leaders because they have the responsibility to maintain the national health systems away from collapsing. We are confident these data could help them into decision-taking and place the capitals (from hospital beds to human resources) into the right place.

## Introduction

The ongoing outbreak of viral pneumonia caused by SARS-CoV-2 (severe acute respiratory syndrome coronavirus 2), globally known as coronavirus disease 2019 (COVID-19), is spreading fast, and it is itself a stress test to the global public health, research, and medical communities. The recent outburst in Italy and Spain along with disease control in China has made the World Health Organization (WHO) establish Europe as the epicenter of the pandemic. A recent study showed that the spread of COVID-19 was unstoppable and has infected more than 1 million people worldwide 150,000 people in 100 countries (1). Today, this number already overpassed a million of deaths cases. COVID-19 is considered as a pandemic threat by WHO (2).

Person-to-person transmission via droplets, contaminated surfaces, or hands has been demonstrated (3) even among asymptomatic carriers to close contacts at mainland China, where the outbreak started in January 2020, and it required of severe population control measures to manage it (4, 5). One month later, the same happened in Italy, with the origin in some imported cases from China. Moreover, 2 weeks later, similar trends emerged in Spain, and the rest of Europe, as well as in the United States, since the beginning due to Asian travelers back and, in the second wave, from European travelers.

The statistical information on the infection pattern happening in China started on January 20, and only 3 days later, Wuhan city was closed in a set of strong measures of isolation, which surprised worldwide. The infection pattern followed in China can be seen in Supplementary Figure 1, and it provided an overview of the number of new cases over the time in China that we used as the reference for modeling.

The spread evolution shows three phases: the first one with a significant and apparent exponential expansion and the second one when a diminishing speed on infection follows until the new numbers are almost zero, whereas the third one is reached when the increase on new infection is almost zero. Since the application of the first containment measures, 12 days were needed to reach the peak of contagious speed (first phase), and from then to the zero growth took another 26 days. The virus outbreak in China concentrated in Wuhan and Hubei province with a relatively few dispersion through the rest of the regions, officially as the result of the isolation measures. These measures could have the effect of changing the trend of the infection series but could not avoid the expansion in the first phase at daily rates of 47.3% on average until February 11. The decreasing rates started on February 12 and reached a daily rate of 0.32% at the beginning of March.

The first significant transmission outbreak in Europe was through Italy. From a few cases imported from China, the virus spread after a few days following similar daily patterns: 40% of new infections on average day-to-day during the first period. An average daily growth rate of 24% was occurring when the government applied harder, containing measures on March 8 (Supplementary Figure 2). In Spain, the infection started as transmitted by leisure and business travelers from Italy. At the beginning of the first phase, the disease seemed to be under control, but it spread from March 4 accelerating from March 8 as a result of the expansion in Madrid. As far as we can tell, the territorial virus outbreak pattern of Spanish was similar to the other two countries, with the main focus concentrated in Madrid and other minor outbreaks in the Basque Country and Catalonia (Supplementary Figure 3).

This article's central hypothesis was that as COVID-19 is a new virus, there was no immunity against it, so it spread out with no restriction among the population if no isolation measures are taken. Moreover, the virus spread did not depend on the healthcare system because the healthcare system was not prepared for its hardness and did not count on preventive elements such as a vaccine. Under the assumption that COVID-19 followed the same pattern as that observed in China, the spillover effect in the infection process should show a similar pattern in all countries with equivalent live and health levels. We focused on how the first contagion spread out last January in China, to define statistically the potential systematic pattern shown and to forecast when and where the process could end after the measures applied by the governments. The systematic updates of data (daily) and the model estimation supported such a hypothesis with substantial evidence in its favor.

The patterns were modeled using signal extraction techniques to forecast the future evolution of the contagious process. Based on those estimations, this article predicted the number of intensive care unit (ICU) needs at the time of writing this article and the public health intensive care requirements due to COVID-19 in Italy and Spain. The predictions made with this model with April data are compared with the real data several months later showing a very precise forecast and supporting this methodology to advance future potential disease evolution and support political decisions.

## Methods and Modeling Predictions

We obtained information on cases with confirmed COVID-19 infection and diagnosis in China, Italy, and Spain based on official reports from Health Minister in the case of Spain (6), from the Italian Department of Civil Protection (7) and the National Health Commission of the People's Republic of China (8). The data were collected in real time each morning, and it may be updated in the afternoon as new cases became publicly available. The latest update to this dataset was on April 6, 2020. Specifically, we collected the dates of accumulated infected people, ICU, and recovered each day.

The analysis of the data that was carried out in this article is purely statistical, based on a well-known signal extraction technic by using ARMA models (9) (Box-Jenkins methodology). It was done in several steps. First, a univariate analysis of the series of infected persons was carried out to identify the autoregressive pattern that shows them. The results revealed a common pattern of disease spread in all three cases in all phases of the contagion. Second, the levels of acceleration were analyzed, and a model was estimated in order to forecast the future infection path in Italy and Spain. The predictive model was estimated first by using an out-of-sample period with high precision and then used to forecast future time. The third model approached the time pattern between the contagious and the number of people in ICU. The infection forecast was used to predict the future needs of ICUs between the two analyzed countries.

This article estimated ARMA models for three countries: China, Italy, and Spain, at an aggregate level to demonstrate the similarities in the contagious pattern (supporting the use of Chinese case as a proxy variable). The reason for choosing China is evident as it was the first country that experienced the pandemic and applied drastic measures for its control, constituting the unique evidence of the process to be considered. Second, Italy was selected because it was the first European country where the disease spread virulently. Its appearance occurred 1 month later than China, and its origin was a result of the contagion of few people traveling between the two countries. The Italian economic center was the first to be affected (Lombardy with Milan followed by Venice) as a clear reflection of the fact that business or leisure global trips on that area contributed to the spread of the disease. Spain was the third country affected as a result of its relationship with Italy. Spain warned of the effect and applied drastic measures starting on March 14.

Moreover, a further model was estimated to predict the ICU beds needed. It is not possible to identify all variables affecting the need for ICU beds (for instance, previous comorbidities, the severity of the symptoms at the onset of COVID-19, the availability of the treatments, among others). Here we used a sophisticated methodology to calculate how the number of infected people could result in ICU need, following both a stable pattern (so-called long-run effect) and unexpected pattern (so-called short-run effect).

The method is known as vector autoregression model (VECM) (10, 11), and it is defined as an econometric tool that would capture the causal relationships among variables and how one influenced others regarding a time lag pattern. The model was able to calculate the total number of beds in ICUs resulting from two sources. The first was the predictable number of beds reflecting the amount of existing cases that could be required (this component is recognized to represent the permanent or stable in the long-run effect in the influences among the variables in a VECM). The second source was an estimation of unexpected extra cases that should attend in ICU departments influenced by COVID-19. These cases may belong to the patients who unexpectedly developed severe complications (this component is the so-called short-run effect). The total number given by the model was the number of beds that should be available at ICU service, taking into account the two mentioned categories. The distinction between both components could be used to forecast the future need for ICUs, which was the aim of this third step.

This research identified the time pattern of the infectious process in Italy and Spain and estimated the number of new cases that could be reached in these countries. Also, we deepened on its implications in healthcare resources devoted to the number of ICU required.

## Results

**Figure 1:**
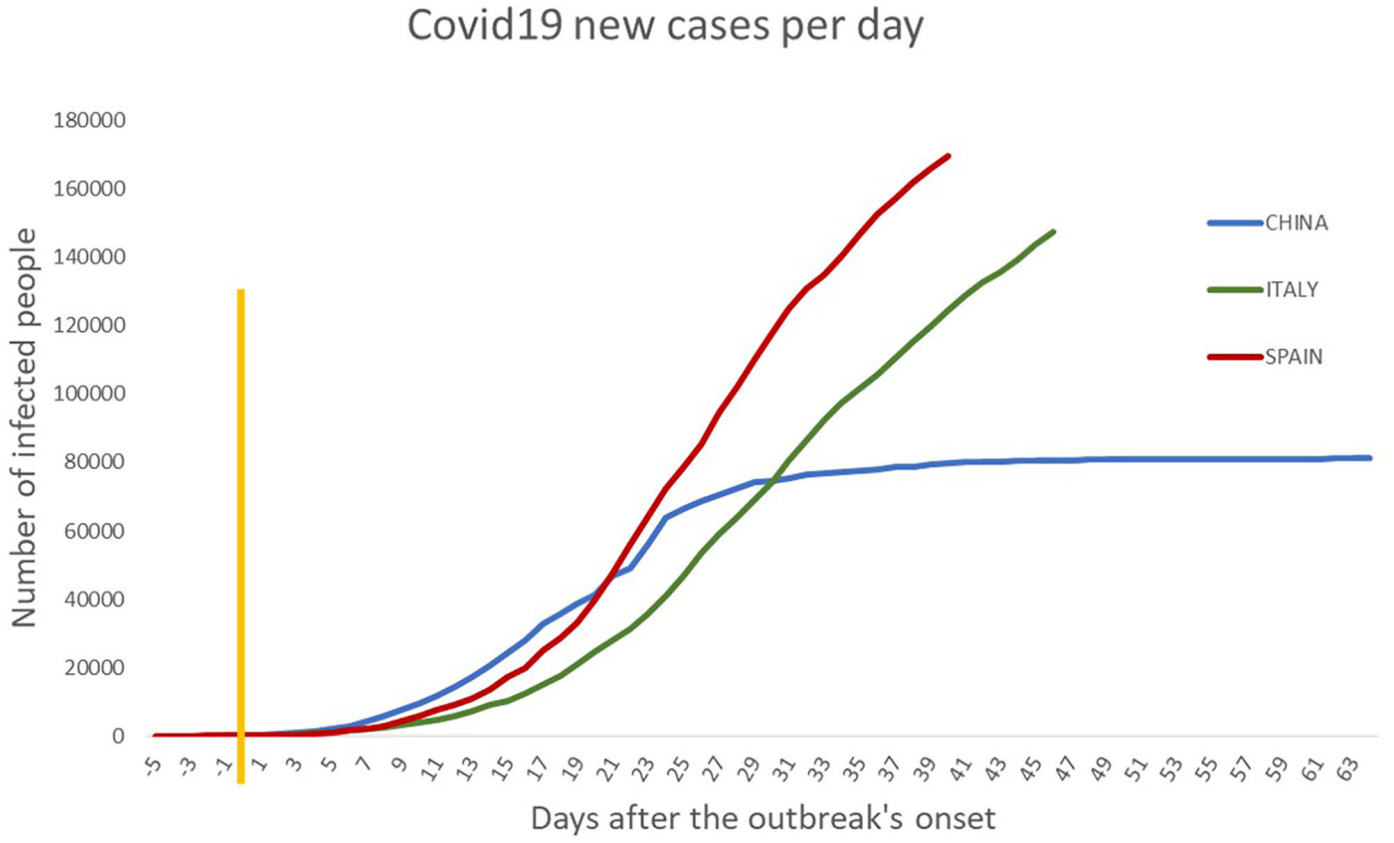
Overview of COVID-19 infection process in China, Spain, and Italy. The numbers of new cases appear on the *Y* axis and the time scale over the *X* axis. The three curves start at the same point since we overlapped them with the aim to see the evolution of the pandemic, starting from the same point (day 0). Specifically, the yellow line represents the “day 0” in which each country had an account for around 200 infected individuals. The final day corresponds to the infection data in early April 2020. The similarity in growth patterns is unique. Such similarity is taken as the base hypothesis in this article through considering that the future evolution of the infection process would be similar in the European countries if the effect of the containing measures has got the same positive impact to that in China.

Before starting with the results from the model, Figure 1 is useful as an overview of the number of new cases (*Y* axis) over time (*X* axis) during the period the model was built. Nevertheless, with illustrative purposes, we overlapped the graphs and made a standard “day 0” for the three countries (marked with a yellow line), and from there, we started to count the days.

First, with empirical evidence, the model estimated new infections for every country. With this step, we asked the model to tell us the “story” of what happened in the three countries under study from day 0 to day 45 (in China, since data were available) and until day 20 (in Italy and Spain). The estimated parameters (called AR and MA) are a measure of how the contagion was expanding across the territory, and they are shown in Table 1 when they were significant. If left in blank, the coefficient showed no significant result, and we left intentionally empty the cell for explanation purposes. AR(1) and AR(2) parameters showed the speed of infection, positive in all three countries. Specifically, the model estimated the new infections, taking into account the number of infected people the day before and the trend of the preceding days. Moreover, there was a key difference between AR(1) and AR(2): AR(1) reflected the endogenous rise of the infection in the population, whereas AR(2) showed the rise of the infection affected by external infected people.

**Table 1:** Time autoregressive pattern in COVID-19 infection process in the three countries of study.

	**Spain**			**Italy**			**China**		
**Sample**	**10 days**	**15 days**	**20 days**	**10 days**	**15 days**	**20 days**	**15 days**	**30 days**	**45 days**
AR(1)		1.101 ± 0.361	1.009 ± 0.400		1.573 ± 0.366	1.176 ± 0.274	1.068 ± 0.439	1.285 ± 0.237	1.506 ± 0.345
AR(2)	0.918 ± 0.433			1.4 ± 0.254					
MA(1)	1.604 ± 0.563	−5.90 ± 0.201	−1.970 ± 0.739		−1.095 ± 0.197	−0.695 ± 0.075	0.678 ± 0.278	−0.616 ± 0.244	−0.992 ± 0.3
MA(2)		−0.492 ± 0.242	1.262 ± 0.409	−0.981 ± 0.219	1.151 ± 0.203	−0.68 ± 0.188		−0.645 ± 0.266	
MA(3)		0.835 ± 0.144	−2.027 ± 0.590			0.874 ± 0.076		0.970 ± 0.115	0.531 ± 0.18

*The estimated parameters (called AR and MA) are a measure of how the contagion expands across the territory. If left in blank, the coefficient shows no statistically significant result, and we left intentionally empty the cell for explanation purposes. AR(1) and AR(2) parameters show the speed of infection, whereas MA parameters reflect the influence on the viral spread of unexpected and nonobservable reasons, like individuals moving across regions not being registered (in the case of positive impact), or the effect of measures applied, like isolation (in the case of negative value)*.

On the other hand, the M.A. parameters reflected the influence on the viral spread of unexpected and nonobservable reasons, such as individuals moving across regions not being registered (in the case of positive impact), or the effect of measures applied, such as isolation (in the case of negative value). The two patterns allowed to discriminate the “direct infection” (with the AR estimated parameters) from the “indirect effect on the contagion” (M.A. parameters).

During the first phase of infection in China, AR(1) values ranged from 1.1 to 1.3, and they did not diminish as days are added in the expanding-windows models. It suggested an intense spread process, dependent only on the number of infected inside the country, which was what happened. It was important to note that China does not show any AR(2) parameter significant, suggesting that no exogenous infection happened in this country. It made sense as the current outbreak of COVID-19 had its origin in that country.

In Italy, during the first phase, the parameter associated with exogenous infection [AR(2), which measured a direct effect from not local infections] suggested that the first cases came from abroad. The value was also explosively capturing the rapid expansion, and on days 15 and 20, Italy showed endogenous expansion from the virus, meaning that the virus dwells within the country. Curiously, this is the same path, followed by Spain. In this country, in the beginning, the infection spread by exogenous factors, and as the series went on in time, endogenous expansion gained strength and explained the current outbreak.

Focusing now on M.A. parameters, there was a positive value of MA(1) in China for the first 15 days, meaning that unknown components were enhancing the infection. The same happened in Spain during the first 11 days when restrictive measures were not taken. Nevertheless, MA(1) turns negative in Italy and Spain on day 15 of the outbreak, possibly capturing how the application of restriction measures contributed to reducing the infection. The same happened with MA(2) parameter: it became negative as the series went on, reflecting external factors that were helping to reduce infection outbursts. The effect of those unknown components was to reduce the spread of the endogenous infection [AR(1)].

In all three cases, there was an extra moving average component [MA(3)] with a positive parameter that acted in the opposite direction of MA(1) and MA(2), representing the power of some factors increasing infection from the past. The value of MA(3) component was very similar during the last period in Italy and Spain, and a bit smaller for China. The interpretation of those results was that the negative M.A.s captured the effect of the locally taken initiatives to deal with the crisis: isolation and restriction. These measures seemed to have had a sudden and rapid impact in all three countries, faster in Spain, with the time pattern changing in only 3 days in an equilibrium manner. In Italy, it took a bit more time to effect until the 20 first days. In China, the broader contention effects seemed to happen since the 30th days (not before) as all previous periods registered time pattern with a positive impact in the infection spread.

**Figure 2:**
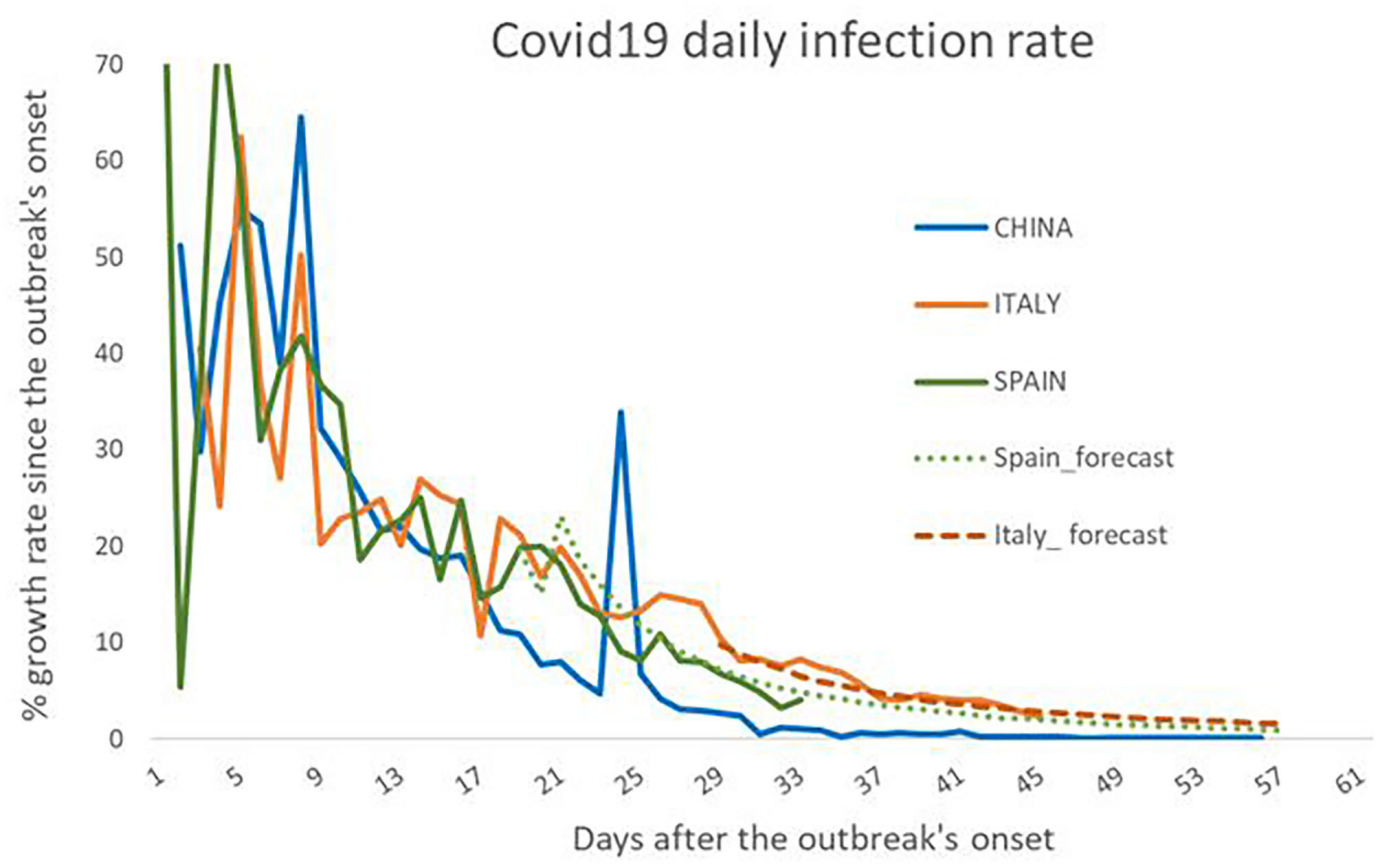
Diary infection's growth rates in percentages for China, Spain, and Italy. The figure represents the daily growth rates (solid lines) for the three countries since the beginning of the outbreak and considering that the start occurs on the same day for three. The model forecast for Italy and Spain is presented through the dotted lines. They show substantial precision in the speed of contagion, suggesting the accuracy of the modelization done.

With the current data, the ARMA model supported that both Spain and Italy followed China's time path, and this could also be shown in Figure 2, representing the daily growth rates. If the first part of the outbreak showed these similarities among countries, we hypothesized that the contagion patterns would have a common time path among the three countries. We assumed that the time pattern found was reliable, and therefore, we think that the event of infection growth could be predicted.

**Figure 3:**
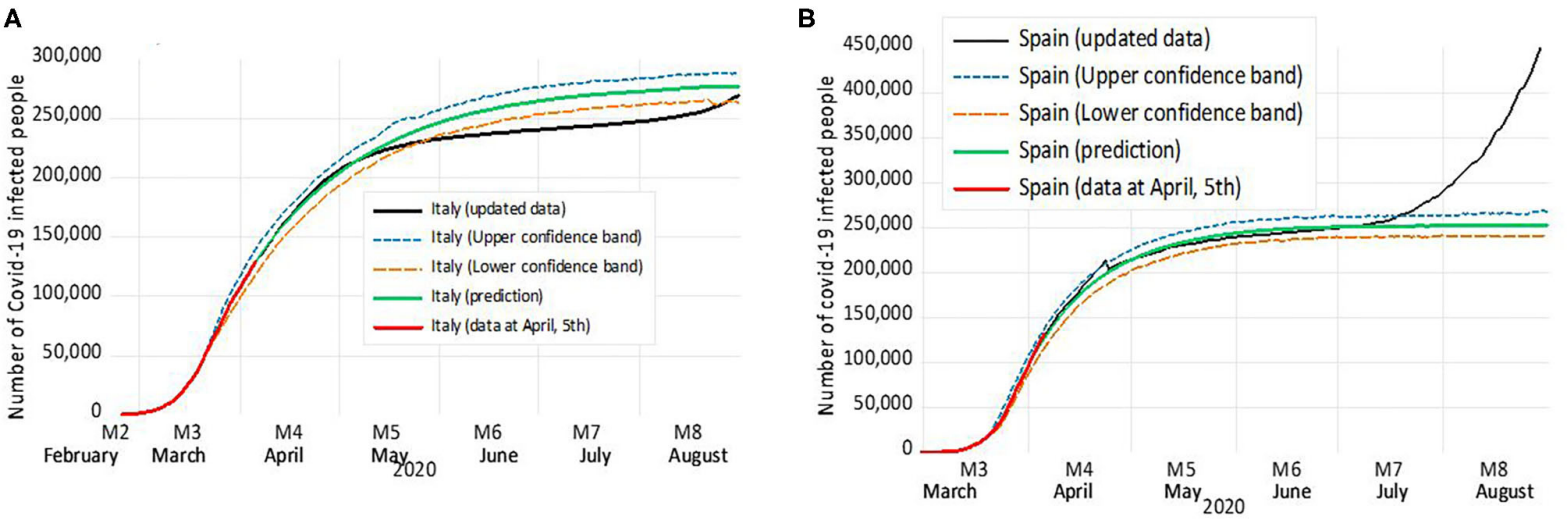
Forecasting for Italy **(A)** and Spain **(B)** according to the ARMA model. In red, we depict each country's real number of cases until April 5 (when the forecast was calculated), whereas in green, we show the prediction according to the model, and in black, the updated data are represented. The 95% confidence margin appears as two lines; the orange dotted line represents the lower confident band, and a blue dotted line represents the upper confident band.

With all the above, a model was defined for forecasting purposes. This model tested the hypothesis that Spanish and Italian's contagion processes followed the path shown in China and adjusted it to their own ARMA pattern. It assumed that there existed a common way of the virus to expand itself adapted to each reality. The results of forecasting for Italy and Spain are shown in Figure 3. We also performed a panel prediction by regions in the case of Italy and by autonomous communities in Spain using the Chinese process as an instrument variable in the autoregressive model to forecast infections (data not shown).

Italy was the European country that first showed a significant infection process and the first where the authorities reacted by applying strong measures of isolation to contain the virus expansion. There was a similar time pattern to the one that happened at the first moments in China, and this further supported the hypothesis of a common global expansion pattern. Besides, Italy showed strongly significant differences by region in terms of the number of infected people, just like China. Specifically, Lombardy, Veneto, Piemonte, and Emilia-Romagna carried a 43.3, 8.6, 8.1, and 13.4%, respectively, of the total number of infected in the country as of March 26. ARMA model predicted a reduction in the contagion speed during the second week of April in Italy and smooth reduction until the peak flattened, with control on new infection happening in most regions including Piamonte and Veneto, but not in Lombardy and Emilia-Romagna. The real attenuation was faster than the one estimated by the model since in mid-May, Italy was in the middle of strong containing measures.

Spain started later the contagion process, and it evolved quickly. Results reflected a precise association with the Chinese pattern during the first phase of the infections as the Spanish process seemed to reach the turning point experienced by China in a similar moment. Just like Italy and China, in Spain, some regions showed a stronger relationship with the global infection pattern. In this case, Madrid and Catalonia had around 30 and 20%, respectively, of all the infected. In the case of Spain, the model predicted that infections still could grow fast until early May, and at that moment, the speed of contagion would be reduced.

Regarding the prediction until the actual data, the maximum level of contagion in Spain surpassed that in Italy because of faster growth in the Spanish number of cases. The precision of this comparison depends on the moment when the infection rate reached the turning point (observed in Italy at the end of March and in Spain early April), and also on the reducing infection speed. The latter relied on success on the isolation and other public measures taken by authorities and fulfilled by population. Note that Italy applied isolation only for Lombardy on March 1st after successive measures of contention since February 25, whereas Spain on March 13 for all the territory and nonessential activities. The updated information for Spain provides a high precision forecast until mid-July (4 full months). From this date onward, Spain has started a new growth in the infection process, enhanced by the ending of confinement measures.

On the other hand, the VECM model estimated different ICU resources in Spain and Italy. First, we showed that the relationship between the total numbers of ICU beds expected due to the affected population size was statistically significant, showing different proportions. The model estimated that 4.2% of total infected by COVID-19 would require a bed in ICUs in Spain the next day, whereas in Italy, this percentage was lower (2%), shown in Table 2. The difference was relevant, and it suggested more challenging implications of the disease in Spain than in Italy, as, with a similar number of infected people in both countries, there was a more intensive use of the ICU in Spain than in Italy.

**Table 2:** Vector autocorrection model (VECM) of ICU requirements for the infection process of COVID-19 in Italy and Spain.

	**Spain**	**Italy**
Sample period, until 4/5/2020, starting date	03/09/2020	03/12/2020
Dependent variable: changes on no. of ICU used beds Variables	Coef	Coef
No. of infected people (−1)	0.042^***^	0.02^***^
(standard error)	(0.012)	(0.003)
c	817.7	—
Convergence parameter of speed to equilibrium	−0.307^**^	0.013
**Short-term sensibility:**		
D [infected people (−1)]	0.014	−0.0343^***^
(standard error)	(0.025)	(0.0136)
D [infected people (−2)]	0.021	0.0344^***^
(standard error)	(0.029)	(0.0169)
D [infected people (−3)]	0.012	−0.016
(standard error)	(0.029)	(0.0142)
Global model tests		
*R*^2^	0.816	0.873
Log likelihood	−160,075	−184,816
**Maximum ICU beds**	**4,068**	**7,843**^**+**^
Date for the peak of ICU beds	April 4	April 19

+ *Estimated*.

*** *p-value < 0.01*,

** *p-value < 0.05*.

*This table contains selected results from a VEC Model estimated with four lags for Spain and three lags for Italy relating the changes in ICU bed requirement associated with an increase in infected people. The cointegration parameter and short-term parameters showing the relationship between ICU and infection are presented. Full results are available under request*.

Regarding the “unexpected” effects, their relevance was captured by the short-term sensibility parameters, which measured the speed at which the new contagions determined the ICU requirements unexpectedly. The model results gave no significant parameters for Spain suggesting that short-term shocks did not determine the ICU needs; in the case of Italy, all parameters were statistically significant, showing that ICU needs in Italy were unexpected in a large number and associated to registered cases at any time.

Besides, the component calculated as “convergence parameter” (part of the VEC model) referred to the capacity of the “stable” number of infections to explain the new ICU numbers. The fact that such parameter was statistically significant for Spain but not for Italy suggested that the ICU requirements in Spain could depend on the new number cases but not in Italy. Therefore, the Spanish ICU needs could be foreseen through the stable relationship between cases and ICU beds and did not depend on some unexpected shock. On the contrary, in Italy, it seemed that the “stable proportion” was not affecting so much the total ICUs, and the evolution of its number depended on several unexpected and short-run-effect events. Those results were supported by the strong significance of the short-term parameters of the newly infected people in Italy, given by the VECM model.

**Figure 4:**
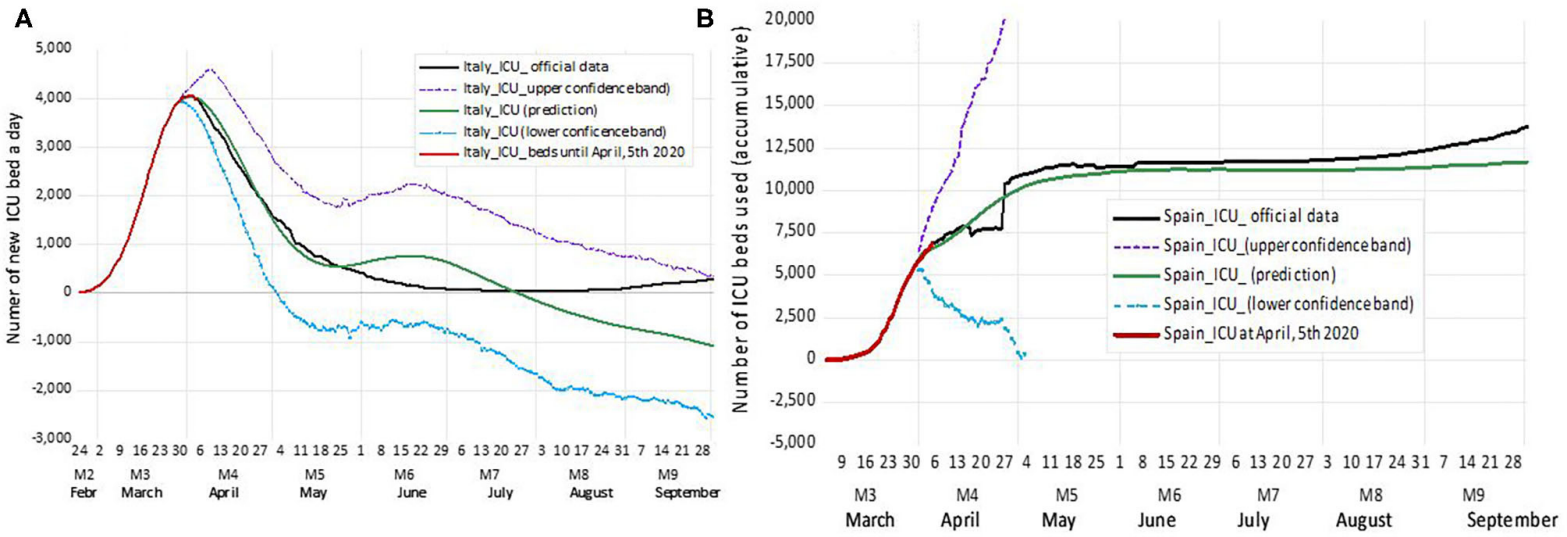
ICU Forecast for Italy **(A)** and Spain **(B)** according to the VECM model. In red, we depict each country's ICU beds in use when the model was estimated (April 5), whereas in green, we show the prediction according to the model. The 95% confidence margin appears as two lines: the blue dotted line represents the lower confident band, and a purple dotted line represents the upper confident band. In black appear the data updated in October 2020 for comparison purposes after the original estimation. The precision in the trajectory and the values, longer for Spain (the model forecast ICU needs with high precision until mid-August) and shorter for Italy (the forecast is highly precise until May 25 and after it predicts a rebound, which has not taken place).

Prediction of ICU needs is shown in Figure 4 and displayed how the Spanish ICU would increase until needing around 8,000 units by the end of April 2020 and rises until 11,000 units. Here the prediction remains stable. On the contrary, the prediction for Italy shows a decrease in the number of ICUs after an initial peak. In the latter case, the model predicted an unexpected rise in ICU needs that did not take place. We think this never happened thanks to the ongoing confinement measures applied during that month (also captured in the forecast of infection process model).

On the other hand, the prediction for Spain was highly precise with the needs of ICU beds, and it shows almost constant until September. This result may seem odd, but it is explained by the fact that the Spanish ICU data correspond to the accumulated number of beds. Therefore, the interpretation is that there is not an increase in ICU demand in this country until September. Instead, the data from Italy refer to new beds required each day, so the interpretation we think is the one above. Note that the increase in the use of intensive care beds in both countries after summer shows a lower speed than at the beginning of infection, reflecting new variables playing a role in the disease control.

In summary, the interpretation of the VECM results suggested that the Spanish COVID-19–infected people were associated with more ICU care in a very stable way, whereas in Italy, the infected people required less coverage immediately. Nevertheless, in this country, the number of ICU needed could rise unexpectedly. If so, the Spanish health system made a significant effort to prepare the ICU services than in Italy, but the latter had the risk to experience a sharp rise in the ICU needs unexpectedly and faced the lack of resources. The application of new contention measures reduced such risk, fortunately, during May in Italy.

## Discussion

This article shows a similar behavior of the infection in all the studied countries, and based on this fact, we use ARMA and VECM methodologies to predict the status quo curve for COVID-19 infection and ICU bed requirement. The results show that with the restrictive measures, we estimate around 180,000 cases for Italy during April and around 240,000 cases in the maximum peak of the curve, and the numbers for Spain are similar, although reached earlier than in Italy. This prediction is based on research tools to give an idea of what might be the damage to the healthcare system or the load burden in a second wave. The infection process requires around 4,000 ICU units in Italy to be available to cover the COVID-19 needs, but they could be more due to the volatile dynamics.

Regarding Spain, we estimate 220,000 cases by mid-April and 250,000 in the maximum peak of the curve. We also estimate a need of around 7,500 ICU beds in Spain on average (although the accumulated final use was around 11,000 beds), as a peak in both cases.

Fortunately, not all the infected cases involve urgent medical assistance, such as an ICU, and this is the crucial point to maintain the most rigid restrictions on the population's mobility. Regardless of the numbers being handled, perhaps this prediction can help the authorities to make better decisions on how to manage the available resources because it gives an idea of what could happen in the future.

We acknowledge the existence of underdiagnosis that may affect the data provided by authorities. Diagnostic precision depends on the number of COVID-19 detection tests, their accuracy, and the way the sample is taken. In Spain, as our knowledge goes, COVID-19 detection tests were made only in people admitted into the hospitals and their close contacts during the first COVID-19 wave. Therefore, the real number of cases could be higher than the ones we estimate in this study on the first wave of the pandemic. This fact does not affect the model estimation as the observations are representative of the whole pattern. Besides, the number of ICU is the total number of used resources (beds), which guarantee more precision in forecasting these variables, as there was no underdiagnosis in this case. In this country, by the end of June and with the aim of controlling small outbreaks, the use of test and contact trackers was implemented in all the territory. As a result, asymptomatic people were detected and included in the statistical data. We think this would be one of the reasons to understand the sharp increase in infection shown in Figure 4 for Spain.

Also, this prediction was made while the confinement measures in Italy and Spain were happening; thus, the number could vary (increase) if people could move freely and face the risk of new exposure to the virus or the measures become greater restrictive. Our model could be refined if we consider the differences between the actual scenarios in Italy and Spain, as, despite looking alike, they are not the same. For example, the characteristics of the population (mean age, disease profiles) or the political, social, or socioeconomic profiles could affect the community's behavior and willingness to accept and follow the rules. There are also differences between the two healthcare systems, which have not been considered in this study. For example, in Spain, besides the several years of low finance support of the healthcare system, the responsibility for health is devolved to 17 very diverse regions, and the coordination of all of them depends on the central government. The effectiveness of the two healthcare systems to detect and treat the disease is different, but we think that the total numbers of cases provided by authorities surpass these limitations and could barely affect our model.

Moreover, the time patterns estimated do capture some of the differences the referee mentions in a lower extent in the infection process but much more clearly in the ICU requirements. For instance, Table 2 shows the time pattern for ICUs needs in Spain and Italy. Spain shows a fast convergence in ICU requirement with the total number of infected people. It means that an increase in infection is immediately transmitted to the need of ICUs; there is no statistically significant lag, which suggests that infected people go to the hospital straight ahead. It is not the case in Italy. The lagged parameter shows half of effected than in Spain, suggesting that less infected people go to the hospital at the very beginning, but they attend later on, as the lagged parameter confirms. These differences in time pattern may reflect, from our understanding, a difference in the population behavior or healthcare structure between both countries, which should be analyzed deeper when more information is available.

Finally, this estimation has been obtained in developed countries that applied efficient contention measures in a very short time; thus, we fear the hardness of the outbreak in less developed countries with fewer resources or in cases where restriction measures are not taken. There will be a constant increase in the number of cases in the following weeks, and all the healthcare systems must be ready to take the lead, as it has already happened in China, Italy, and now Spain. We encourage all the authorities to take strong measures to minimize the effect of the outbreak in their countries, such as social distancing, forbid people's movement, and promote basic preventive measures such as hand washing.

## Data Availability Statement

Publicly available datasets were analyzed in this study. This data can be found here: https://www.mscbs.gob.es; http://www.nhc.gov.cn; http://www.salute.gov.it/portale/home.html.

## Author Contributions

PM, ZS, and LG were responsible for data recollecting. PM did the data analysis and statistical models. PM and PT wrote the manuscript. All authors contributed to the article and approved the submitted version.

## Conflict of Interest

The authors declare that the research was conducted in the absence of any commercial or financial relationships that could be construed as a potential conflict of interest.

## References

[1] Callaway E Time to use the p-word? Coronavirus enter dangerous new phase. Nature. (2020) 579:10–38. 10.1038/d41586-020-00551-1 33623145

[2] WHO Director-General's Opening Remarks at the Media Briefing on COVID-19 Available online at: https://www.who.int/dg/speeches/detail/who-director-general-s-opening-remarks-at-the-media-briefing-on-covid-19-−11-march-2020 (accessed 12 March 2020).

[3] Paules CI Marston HD Fauci AS. Coronavirus infections—more than just the common cold. JAMA. (2020) 323:707–8. 10.1001/jama.2020.0757 31971553

[4] Bai Y Yao L Wei T Tian F Jin DY Chen L. Presumed asymptomatic carrier transmission of COVID-19. JAMA. (2020) 323:1406–7. 10.1001/jama.2020.2565 32083643 PMC7042844

[5] Li Q Guan X Wu P Wang X Zhou L Tong Y. Early transmission dynamics in Wuhan, China, of novel coronavirus–infected pneumonia. N Engl J Med. (2020). 382:1199–207. 10.1056/NEJMoa2001316 31995857 PMC7121484

[6] Italian Ministerio della Salute Covid-19 Bollettino, Protezione Civile. Available online at: http://www.salute.gov.it/portale/home.html (accessed October 4, 2020).

[7] Spanish Ministry of Sanidad Available online at: https://www.mscbs.gob.es/~profesionales/saludPublica/ccayes/alertasActual/ (accessed October 4, 2020).

[8] National Health Commission of the People's Republic of China (中华人民共和国国家卫生健康委员会) Available online at: http://www.nhc.gov.cn/ and http://en.nhc.gov.cn/DailyBriefing.html (English version). (accessed October 4, 2020).

[9] Box GE Jenkins GM Bacon DW Models for forecasting seasonal and non-seasonal time series. In Harris, B, editor. Spectral Analysis of Time Series. New York, NY: John Wiley and Sons, Inc (1967).

[10] Johansen S Likelihood-Based Inference in Cointegrated Vector Autoregressive Models. Oxford: Oxford University Press (1995)

[11] Johansen S Interpretation of cointegrating coefficients in the cointegrated vector autoregressive model. Oxf Bull Econ Stat. (2005) 67:93–104. 10.1111/j.1468-0084.2005.00111.x

