# Supplementary material for: COVID-19 Infection Process in Italy and Spain: Are Data Talking? Evidence From ARMA and Vector Autoregression Models
Source: Front Public Health. 2020 Nov 23;8:550602. doi: 10.3389/fpubh.2020.550602 (PMC7719814; doi:10.3389/fpubh.2020.550602)
Supplement: Supplementary file 1 [file Data_Sheet_1.PDF]

SUPLEMENTARY FIGURES

Suplementary Figure-1. Covid19 cases evolution in China: Jan 20th to March 22th

Panel A | Panel B

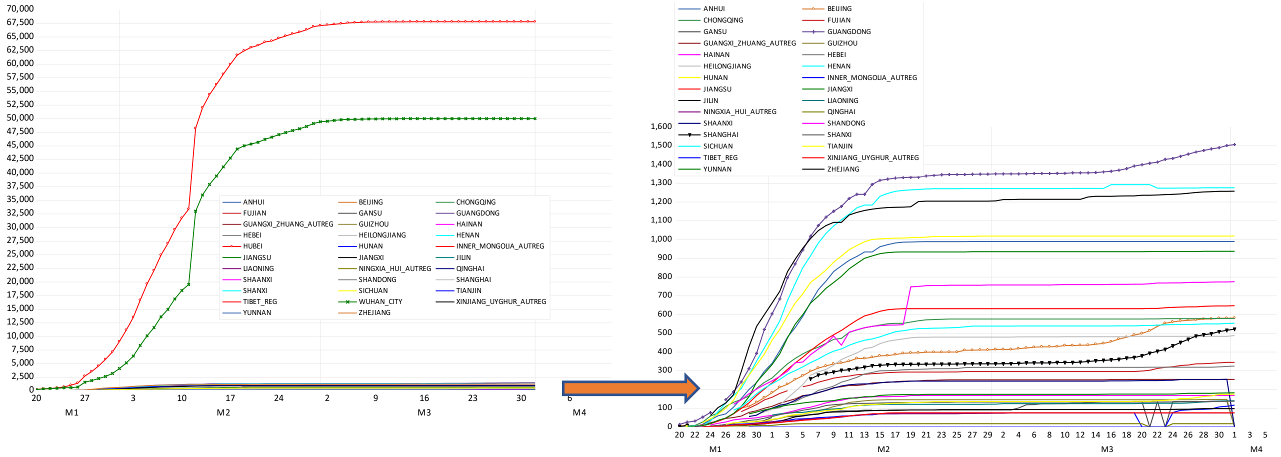

Source: 中华人民共和国国家卫生健康委员会, National Health Commission of the People's Republic of China, http://www.nhc.gov.cn/ , English version web: http://en.nhc.gov.cn/DailyBriefing.html

Suplementary - Figure 2. Covid19 infection pattern in Italy: Feb 28th to March 26th

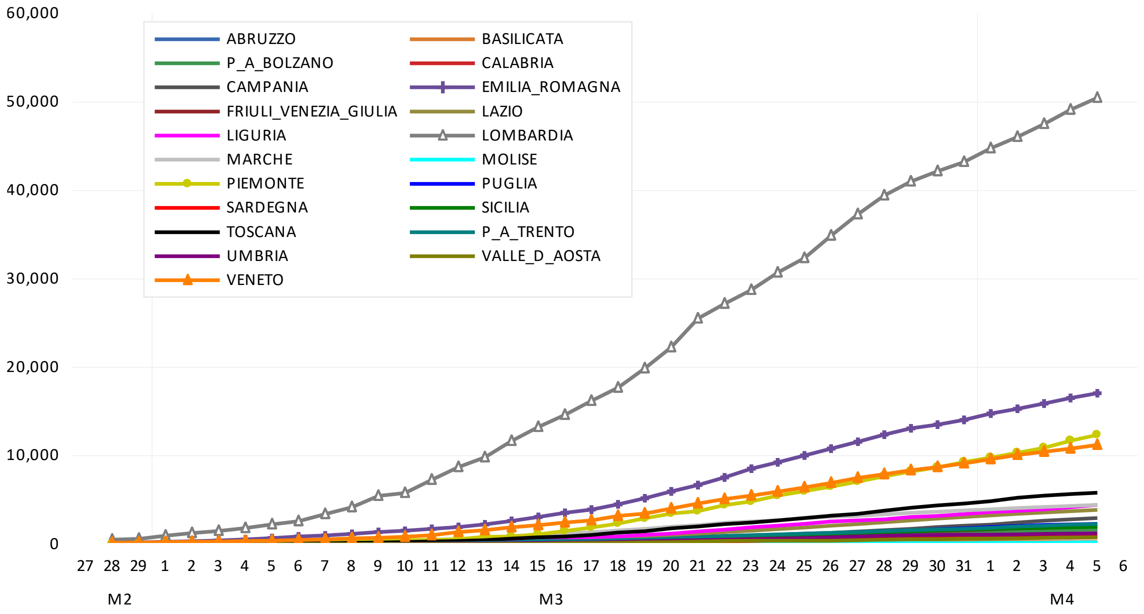

Source: Bollettino Covid-19, Protezione Civile, www.salute.gov.it/portale/news. Several days

Suplementary Figure 3. Covid19 infection pattern in Spain: March, 3rd to 26th

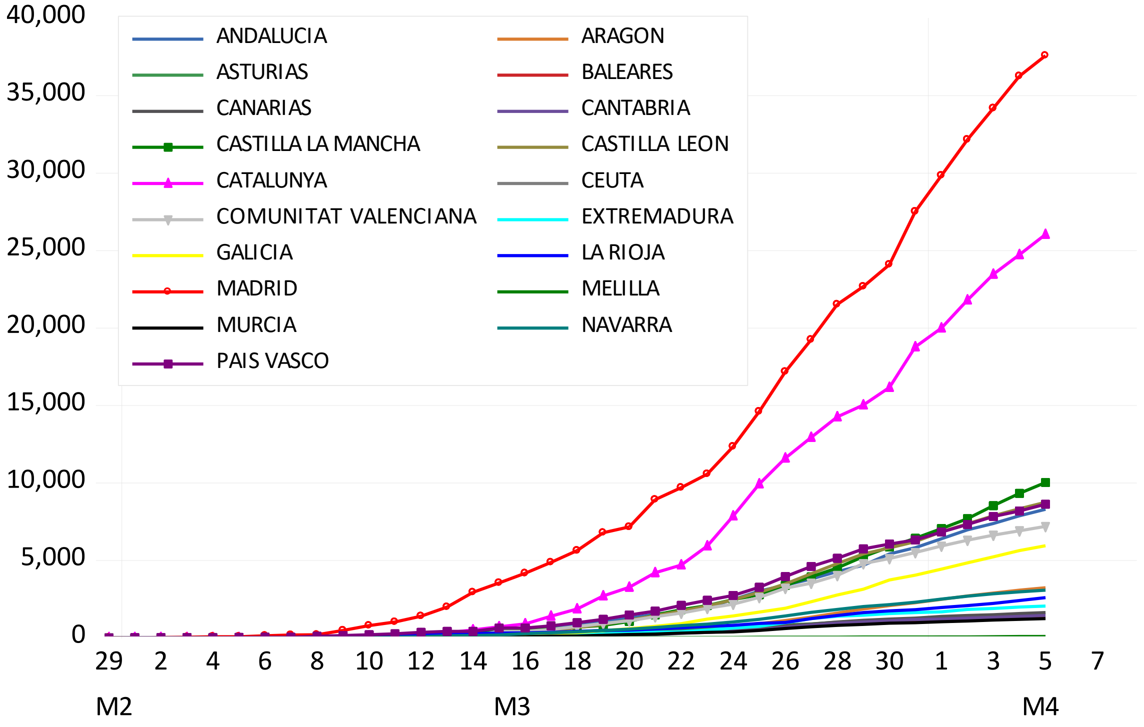

Source: Ministerio de Sanidad, https://www.mscbs.gob.es/profesionales/saludPublica/ccayes/alertasActual/
Several days
